# Validation of the Questionnaire to Identify Knee Symptoms (QuIKS) using Rasch analysis

**DOI:** 10.1186/s12955-015-0358-6

**Published:** 2015-09-29

**Authors:** Clayon B. Hamilton, Monica R. Maly, J. Robert Giffin, Jessica M. Clark, Mark Speechley, Robert J. Petrella, Bert M. Chesworth

**Affiliations:** Graduate Program in Health and Rehabilitation Sciences, Western University, London, ON Canada; Institute for Applied Health Sciences, McMaster University, Hamilton, ON Canada; Department of Surgery, Schulich School of Medicine and Dentistry, Western University, London, ON Canada; Department of Surgery, University of Alberta, Edmonton, AB Canada; Department of Epidemiology and Biostatistics, Schulich School of Medicine & Dentistry; Schulich Interfaculty Program in Public Health, Western University, London, ON Canada; Department of Family Medicine, Schulich School of Medicine and Dentistry, Western University, London, ON Canada; School of Physical Therapy and Department of Epidemiology and Biostatistics, Schulich School of Medicine & Dentistry, Western University, London, ON N6G1H1 Canada

**Keywords:** Outcome assessment, Knee osteoarthritis, Questionnaire, Reliability and validity, Knee pain, Lived experience

## Abstract

**Background:**

The Questionnaire to Identify Knee Symptoms (QuIKS) was recently developed to promote activity by screening for experiences related to early symptoms in people with emergent chronic knee pain problems, such as osteoarthritis (OA) – like knee pain. The main purpose of the current study was to evaluate measurement properties of the QuIKS using Rasch analysis in a sample of people with knee symptoms consistent with symptomatic knee OA.

**Method:**

This study used cross-sectional data. The sample was 200 subjects along the following knee health continuum: pain-free healthy knees (*n* = 55) from a university community, knee pain with no knee OA diagnosis (*n* = 111) from a university-affiliated medical clinic, and patients with surgeon-diagnosed symptomatic knee OA awaiting high tibial osteotomy (*n* = 34) from a sports medicine surgical clinic. The 13-item QuIKS was evaluated for its factor structure, item- and person-fit, item’s category response structure, differential item functioning by sex and obesity status, local item dependency, unidimensionality, and test precision. Subsequently, the QuIKS underwent known-groups analysis and convergent validity with the Knee injury and Osteoarthritis Outcome Score (KOOS).

**Results:**

In the QuIKS, each item’s category response structure was modified. No differential item functioning was observed. Local item dependency informed the formation of four testlets. This refined QuIKS obtained summary fit to the Rasch measurement model, unidimensionality, reliability (person separation index = 0.82), and interval-level scoring. Subsequently, the Rasch-validated QuIKS (QuIKS-R) demonstrated excellent known-groups validity and good convergent validity with the KOOS (Spearman’s rho = 0.45 to 0.77).

**Conclusions:**

The QuIKS-R provides interval-level quantification of knee symptoms-related experiences in people with knee symptoms consistent with symptomatic knee OA. Its scores might be useful for clinicians for promoting activity in individuals with early symptoms consistent with symptomatic knee OA.

**Electronic supplementary material:**

The online version of this article (doi:10.1186/s12955-015-0358-6) contains supplementary material, which is available to authorized users.

## Introduction

Symptomatic knee osteoarthritis (OA) is a chronic degenerative joint disease in which knee pain and changes in the joint structure are related to ill-effects that include physical impairments, activity limitations, participation restrictions, and reduced quality of life [1–4]. In the United States of America, the lifetime risk of developing symptomatic knee OA is up to 23.9 %, depending on one’s sex, age, and obesity status [3]. The lived experience of people with knee OA is considered biopsychosocial, and is associated with the ill-effects of the condition [4–8]. Furthermore, measurement of these experiences might be useful in identifying people with early stage knee OA symptoms for therapy, because studies have shown that during the pre-diagnosis stage and early stages of knee OA as well as when symptomatic knee OA is recently diagnosed, people appraise, perceive, form intentions around, and respond to their knee symptoms in certain ways [4–8].

One measure, the Questionnaire to Identify Knee Symptoms (QuIKS), was specifically developed for clinical and research use “to identify emerging knee problems in people who could benefit from conservative interventions” (p. 1) by quantifying patient’s experiences [9]. The QuIKS is a 13-item self-administered discriminative questionnaire [9]. It was developed using a mixed-methods approach, which aligns with recommendations for scale development by Velozo and colleagues [10]. First, its items were generated through qualitative research that used grounded theory to describe a process of how people with knee symptoms go through a cycle of perceiving, forming intentions, and exhibiting behaviours directed at preventing damage when engaged in physical activity [5]. This was followed by rheumatology experts’ consensus, then item reduction and internal consistency evaluation [9]. However, construct validation has not been performed for QuIKS. Also, Velozo and colleagues recommended that Rasch analysis should be used to determine whether a measure captures a unidimensional construct, which is a form of construct validation [10]. This last recommendation was the main purpose of this paper.

In Rasch analysis, observed data are expected to fit the probabilistic relationship within and between person estimates and item estimates as specified in the Rasch measurement model [11, 12]. Consequently, a questionnaire with data that fits the Rasch model has a unidimensional construct, thereby having interval-level measurement properties as recommended for questionnaires used as measures [10, 13, 14]. Importantly, compared to ordinal-level or nominal-level summed scores, interval-level measurement properties allow for making more accurate interpretations on the relative distance between scores on the scale of a measure [12].

The primary purpose of this study was to evaluate the factor structure, items’ category response structure, item- and person-fit, differential item functioning, local item dependence, overall fit, unidimensionality, and test precision of the QuIKS using Rasch analysis in a sample of people with knee symptoms consistent with symptomatic knee OA. Our secondary purpose was to subsequently evaluate the known-groups validity and the convergent validity of the Rasch-validated QuIKS using the same sample.

## Methods

### Design

This study used cross-sectional data. We recruited subjects into three distinct groups along the following knee health continuum: pain-free healthy knees (HK), knee pain with no knee OA diagnosis (KP), and surgeon-diagnosed knee OA scheduled for high tibial osteotomy (pre-HTO). Subjects in the HK group self-reported no knee pain in the past three years and were between the ages of 20 to 40 years. Subjects in the KP group had verbally complained of knee pain lasting two or more weeks to their family physician within the previous three years as recorded in their medical chart and were between the ages of 40 and 65 years. Subjects in the pre-HTO group were between the ages of 40 and 65 years. The HK group was recruited (March 2011 to January 2012) from a university community through posted paper notices. The KP group was retrospectively collected from data collected (April to August 2009) through a university-affiliated medical clinic using mailed questionnaires as previously described in the publication on the development of the original QuIKS which used some of this data [9]. The pre-HTO group was prospectively collected (March 2011 to January 2012) through a university-affiliated sports medicine clinic using mailed questionnaires. Each subject had to be able to read and understand English to participate in this study. We excluded persons with gout, rheumatoid arthritis, chronic low back pain, foot or hip pain, major co-morbidities, previous knee arthroplasty, or high tibial osteotomy. These exclusion criteria helped to ensure that the knee pain and the illness experiences of subjects were consistent with symptomatic knee OA. Ethics approval was granted by Western University’s Health Sciences Research Ethics Board. Each participant provided written informed consent.

### Participants

The total sample was 200 subjects along the knee health continuum. The HK, KP, and pre-HTO group had 55, 111, and 34 subjects, respectively.

### Outcome measures

The sample descriptive data included sex, age, body mass index (BMI), affected knee (unilateral, bilateral, or none), family history of arthritis (yes or no), and history of knee injury (yes or no). To indicate the structural severity of knee OA, a single rater recorded the Kellgren and Lawrence grade from standard weight-bearing radiographs of each symptomatic knee in the pre-HTO group [15]. A Kellgren and Lawrence grade of 0, 1, 2, 3, and 4, represented normal, doubtful, minimal, moderate, and severe knee (tibiofemoral) OA, respectively [15].

### The QuIKS

We analyzed the QuIKS, but data were collected on its 35-item prototype questionnaire, as in the initial validation of the questionnaire, to allow for consistency of data collection across the study groups [9]. The QuIKS has 13 items and four subscales, and each item has a 5-point rating scale. Some items use an adjectival scale to quantify frequency (0 = never, 4 = always), while others use Likert responses from strongly disagree (0) to strongly agree (4). The 3-item medication subscale captures medication usage to relieve knee pain. The 3-item monitoring subscale captures a person’s awareness of their knee symptoms. The 4-item interpreting subscale captures one’s understanding of their symptoms. The 3-item modifying subscale captures an individual’s changes or intention to change engagement in activity in order to avoid progressive knee damage. Since each subscale may operationalize aspects of the lived experience associated with early symptoms consistent with knee OA, combining these subscales into a single measure might reflect a higher-order construct of these experiences. This higher-order construct would be expected to be unidimensional. When normalized, the summative total scores of the subscales of the QuIKS vary from 0 to 100 (worst to best state).

### The KOOS

The Knee injury and Osteoarthritis Outcome Score (KOOS) is a 42-item knee-specific self-administered questionnaire [16]. It captures health status in the following five subscales: pain, other symptoms, activities of daily living, sport and recreation function, and knee-related quality of life [16]. The total scores of each subscale were normalized to a 0 to 100 (extreme to no problems) scale. The KOOS has been widely used and has demonstrated validity, reliability and responsiveness for adults of all ages with acute and chronic knee pain problems [17, 18]. The KOOS was chosen to demonstrate convergent validity of the QuIKS because both measures have a similar target population. However, the KOOS evaluates symptoms severity, physical function, activity in daily living, and quality of life, whereas the QuIKS evaluates experiences associated with these symptoms.

### Data analysis

#### Sample characteristics

Descriptive characteristics were summarized for the knee health groups. The Shapiro-Wilk test evaluated normality of the data within each group of knee health. Factor analysis and Rasch analysis used only the KP and pre-HTO groups combined (*n* = 145), because scores within the HK group were extreme and would not contribute to these analyses. Data analyses were performed with SPSS version 20.0 (SPSS Inc, Chicago, Illinois), or other specialized software as stated in the upcoming sections.

### Factor analysis

As recommended by Tennant and Pallant [19], Horn’s parallel analysis was performed to determine the number of factors to extract from the QuIKS prior to its Rasch analysis [19, 20]. This determined whether the QuIKS had only a single dominant construct as required for proceeding to Rasch analysis [19]. A minimum sample size requirement of 130 participants was calculated using the 10:1 subject-to-variable rule [21]. Horn’s parallel analysis used principal components analysis (PCA) with Monte Carlo simulation to determine the number of factors in the QuIKS’s data. This was done by identifying the number of factors with an empirical eigenvalue, including their 95 % confidence intervals (CI), that were greater than the corresponding eigenvalue generated from 1000 random datasets at a 95 % confidence level [20]. Horn’s parallel analysis is more accurate than other forms of factor analysis, such as the eigenvalues-greater-than-one rule and the scree plot [20]. The 95 % CI of the empirical eigenvalue for each factor was calculated using a formula published elsewhere [22]. Following parallel analysis, PCA with varimax rotation determined the percentage variance explained by each factor.

### Rasch analysis

Rasch analysis evaluated the fit of the data collected by the QuIKS to the Rasch model [23, 24]. The RUMM2030 software (RUMM Laboratories, Perth, Australia) was used, which is a sophisticated and widely used software that is specialized for Rasch analysis. An estimated minimum sample size of 144 subjects was adequate for Rasch analysis for items calibration with ± 0.05 logits at 95 % confidence even if the scale is poorly targeted [25]. However a minimum sample size of 100 subjects is considered to be adequate in most cases at this confidence level [25].

We hypothesized that the QuIKS would contain a unidimensional dominant construct. We used the following 12 steps and previously published fit criteria for the Rasch model to investigate this hypothesis [24]. Step 1: to evaluate goodness-of-fit, the data were divided into two class intervals using the subjects’ total scores. Step 2: a Fishers Likelihood test was performed. If significant (*P* < 0.05 with Bonferroni correction for the number of items), it suggested that the partial credit model version of the Rasch model should be used [26]. Step 3: data of misfitted subjects, those with residual values outside ±2.5, were removed to allow for accurate estimation of the questionnaire’s measurement properties. Step 4: response categories were expected to be sequentially ordered. Disorder occurred when any response category of an item always had less than 50 % probability of being endorsed when compared to each adjacent response category. When disordered response categories were identified, the response structure of the rating scale was corrected by combining two or more adjacent response categories. Step 5: the fit of each item was evaluated. An items misfitted the model if its residual value was above +2.5 and/or had a significant chi-square (*χ*^2^, *P* < 0.05 with Bonferroni correction for the number of items in the questionnaire). Any misfitted item was deleted because it did not align with the construct captured collectively by the other items. All the preceding steps were iterative.

Step 6: the remaining data were evaluated for summary fit to the Rasch model as defined by a non-significant item-trait interactive *χ*^2^ (*P* < 0.05 with Bonferroni correction), mean person- and mean item- residual value (standard deviation) of ~0 (~1). Step 7: each item was examined for differential item functioning (DIF) across two subject characteristics considered clinically relevant to the experiences associated with knee symptoms: sex (male/female) and body mass index (i.e., BMI cut point obese [≥30 kg/m^2^]/not obese) using two separate two-way analysis of variance (ANOVA) procedures. In each two-way ANOVA, the two independent variables were the subjects’ overall construct estimate divided into two class intervals and a subject characteristic. Each item had one mean score for the subjects in each class interval which formed the dependent variable. An item with DIF does not provide consistent estimation of the construct across the categories of the subject characteristics for subjects with equal overall estimates [24]. Step 8: item pairs with their residual correlation > 0.2 after mathematically removing the dominant construct, were considered to have displayed local item dependency, which means that those items were associated beyond the dominant construct in the questionnaire [27]. Such items were combined into a testlet [27].

A testlet is a group of two or more very closely associated items that give a similar estimate of a subject’s level of the construct. Testlets are sub-constructs of a scale, whereas subscales may or may not be sub-constructs. Step 9: the misfitted subjects’ data (from step 3) were re-entered and the changes to the QuIKS in step 1 to 6 were repeated. This allowed all subjects who fit the Rasch-refined QuIKS to be accounted for in the subsequent steps of Rasch analysis. Step 10: we formally evaluated whether the dominant construct was unidimensional.

Unidimensionality is a vital component for interval-level measurement. In the context of testlets, the construct was the common variance (A) among the testlets [27, 28]. Each subject had an estimate generated for two exclusive sets of items, using the Smith method [29]. The two estimates for each subject were then compared using an independent *t*-test [29].

Unidimensionality was confirmed if less than 5 % of subjects had significant *t*-scores, as estimated by the lower bound of a binomial 95 % CI [24]. Step 11: reliability (or scale precision) was then evaluated using the person separation index (PSI). A PSI value of 0.8 indicated the questionnaire can distinguish subjects in up to three levels of the dominant construct, which is the minimum acceptable level for a measurement scale [30]. Step 12: targeting of the sample by the refined QuIKS was evaluated. This step investigated whether the spectrum of the construct captured by the refined QuIKS covered the spread of the construct in the sample. Ideally, the difficulty thresholds of the items should be adequately spread to capture the quantity of construct in every subject. Statistically, this was indicated by a mean person estimate (standard deviation) of ~1 (~0) when the mean item estimate was zero on the same logit (log-odd units) scale of the dominant construct. Also in this step, the estimate of each testlet was determined. This allowed us to determine the hierarchical order of the testlets on the dominant construct based on their logit scores. Lower logit scores represented the tendency of an item or testlet to capture lower levels of the dominant construct. A floor and ceiling effect was 15 % or more subjects with the maximum or minimum scores, respectively [31]. When the QuIKS was adequately validated by Rasch analysis, we adapted a conversion formula [32], and transformed its summative total raw scores to interval-level scores.

### Confirmatory factory analysis

This was performed to test the factor structure in the Rasch-validated QuIKS. Version 7.3 of the Mplus software (Muthén & Muthén, Los Angeles, California) was used [33]. Total scores were calculated for the Rasch-validated QuIKS to allow for testing if there was a higher-order construct. Model fit was evaluated using the following fit indices and cut-off criteria for adequate fit; comparative fit index (CFI, >0.90), the Tucker-Lewis index (TLI, >0.90), and the root-mean-square error of approximation (RMSEA, <0.08) [34].

### Known-groups analysis

We hypothesized that the total scores from the Rasch-validated QuIKS would be significantly higher for the HK versus the KP group (*n* = 166), and higher for the KP versus the pre-HTO group (*n* = 145) with at least a moderate effect size. The estimated sample size was 52 subjects per group for a moderate effect size [35]. We used the Kruskal-Wallis H test (the non-parametric version of a 1-way ANOVA) with the Mann–Whitney *U* test (the non-parametric version of an independent *t*-test) for post-hoc testing because the data had a non-normal distribution. Effect size (*r*) from the Mann–Whitney test was calculated as *r* = *z*/√*n* and then converted to Cohen’s *d* = 2*r*/√(1 - *r*^2^), where *z* was the *z*-score value obtained from the Mann–Whitney test and *n* was the total sample used in the analysis [36]. A Cohen’s *d* of 0.41 was considered small and the minimum effect size for a clinically relevant effect, 1.15 and ≥2.70 were moderate and strong effects, respectively [37]. The 95 % CI of Cohen’s *d* was calculated as *d* ± 1.96*Standard Error [38].

### Convergent validity

We hypothesized that a similar degree of moderate correlation would be observed between scores on the Rasch-validated QuIKS and each subscale of the KOOS. This hypothesis was based on reasoning that the KOOS subscales should be substantially related to a measure that quantifies experiences related to early symptoms of knee OA. Spearman's rank correlation coefficients (*r*_*s*_) quantified these relationships. The HK group was excluded to prevent errors in *r*_*s*_ that would be caused by these subjects' extreme scores. Moderate correlation of *r*_*s*_ ≥ 0.5 supported convergent validity [39]. This analysis required an estimated sample size of 129 subjects, calculated using *r*_s_ of 0.7 (95 % CI = 0.5, 0.9) at an alpha value of 0.05, which was adequately met by the present study's sample [40].

## Results

### Sample characteristics

Response rate was 63.0 % for the KP and pre-HTO group, and not applicable to the HK group [9]. The sample characteristics are summarized in Table 1. Females were less represented in the pre-HTO group in comparison to the KP group.

**Table 1:** Sample characteristics by study groups

Characteristics	Known groups			
	Healthy knees, *n* = 55	Knee pain, *n* = 111	Knee osteoarthritis (pre-HTO), *n* = 34	Knee pain and pre-HTO, *n* = 145
Age, years				
Mean (SD)	24.7 (4.4)	52.1 (6.8)	48.9 (6.5)	51.3 (6.8)
Sex				
Female (%)	35 (63.6)	62 (55.4)^a^	9 (36.0)	71 (49.0)
BMI, kg/m^2^				
Mean (SD)	22.9 (3.1)	28.1 (9.1)	29.1 (4.7)	28.3 (8.3)
Affected knee				
Unilateral (%)	1 (1.8)	61 (55.0)	18 (52.9)	79 (54.5)
Bilateral (%)	4 (7.3)	49 (44.1)	16 (47.1)	65 (44.8)
None (%)	50 (90.0)	1 (0.9)	0	1 (0.7)
Family history of arthritis				
Yes (%)	23 (42.6)^a^	52 (46.8)^a^	11 (33.3)^a^	63 (43.4)^b^
History of knee injury				
Yes (%)	3 (5.5)	77 (69.4)^c^	23 (71.9)^b^	100 (69.0)^d^
History of knee pain				
Yes (%)	2 (3.6)	51 (45.9)	32 (100)^b^	83 (57.2)^e^
Kellgren and Lawrence Grade, Number of knees with Grade 0/1/2/3/4	--	--	0/10/20/11/4	--
KOOS, range = 0–100 (worst to best state), median (IQR)				
Other symptoms	100 (7.1)	53.6 (19.6)	37.5 (29.5)	53.6 (21.4)
Pain	100 (2.8)	80.6 (27.8)	48.6 (23.6)	72.2 (30.6)
ADL	100 (0)	89.7 (23.2)	58.8 (27.7)	80.9 (29.4)
Sport & Recreation	100 (0)	75.0 (40.0)	17.5 (39.1)	58.0 (50.0)
QOL	100 (0)	68.8 (31.3)	15.6 (31.3)	56.3 (43.8)

Kellgren and Lawrence grade severity: 0 (normal) is no OA; 1 (doubtful) is possible joint space narrowing and osteophytes, 2 (minimal) is definite joint space narrowing and osteophyte, 3 (moderate) is definite joint space narrowing, multiple osteophytes, some sclerosis and possible bone contour deformity, 4 (severe) is marked joint space narrowing, large osteophytes, severe sclerosis and definite bone contour deformity [20]

*BMI* body mass index, *KOOS* knee injury and osteoarthritis outcome score, *ADL* activities of daily living, *QOL* quality of life, *IQR* inter-quartile range

Missing data ^a^ *n* = 1, ^b^ *n* = 3, ^c^ *n* = 4, ^d^ *n* = 9, ^e^ *n* = 2

### Number of factors

Table 2 shows the results of the Horn’s parallel analysis which indicated that only the first factor was suitable for extraction from the QuIKS’s data and accounted for 45.9 % of the total variance in its score. Therefore, the QuIKS contained a single dominant construct.

**Table 2:** Results from factor analysis using horn’s parallel analysis

Factor	Empirical eigenvalue (95 % CI)	Randomly generated eigenvalue	Percent variance explained by empirical eigenvalue
1^a^	5.97 (5.02, 6.92)	1.67	45.9
2	1.35 (1.13, 1.57)	1.49	10.4
3	1.22 (1.03, 1.41)	1.36	9.3
4	1.12 (0.94, 1.30)	1.26	8.6
5	0.69 (0.58, 0.80)	1.18	5.2

95 % CI means 95 % confidence interval

^a^Only factor suitable for extraction from the QuIKS

### Data fit to the Rasch model

Rasch analysis used the partial credit model. The main results of Rasch analysis are summarized in Table 3. Initially, the QuIKS did not fit the Rasch model. Therefore, its measurement properties were refined through eight rounds (runs) of Rasch analysis. One set of modifications or data manipulation was performed in each run of Rasch analysis, guided by information obtained in the preceding runs.

**Table 3:** Summary fit statistics from Rasch analysis

Version	Data changes	Sample size	Item-trait interaction χ^2^		Item fit residual		Person fit residual		PSI	Significant *t*-tests
			Value (*df*)	*P* value	Mean	SD	Mean	SD		
Initial	None	145	73.512 (13)*	0.000	0.49	1.84	−0.22	1.18	0.89	7.0
Run 2	Deleted 8 misfit persons	137	72.550 (13)	0.000	0.43	1.93	−0.14	1.01	0.90	5.2
Run 3	Rescored all misfit items	137	19.693 (13)	0.103	−1.01	1.21	−0.66	1.29	0.89	1.6
Run 4^a^	Deleted 20 misfit persons	117	16.105 (13)	0.243	−0.58	1.12	−0.41	1.08	0.90	4.8
Run 5	Formed 4 testlets	117	0.937 (4)	0.92	0.26	0.72	−0.35	0.87	0.84	1.3
Run 6^a^	Used initial data, rescored all items	145	19.480 (13)	0.108	−1.07	1.33	−0.70	1.33	0.89	4.3
Run 7	Formed the 4 testlets again	145	3.546 (4)	0.47	0.02	0.85	−0.45	0.89	0.83	2.9
Run 8	Deleted 1 misfit persons	144	3.612 (4)	0.46	0.03	0.85	−0.43	0.86	0.82	2.9
Rasch-Refined	Deleted 3 persons with incomplete data	141	3.613 (4)	0.46	0.00	0.87	−0.44	0.86	0.82	3.0

Criteria of fit to Rasch Model: minimum sample size of *n* = 108, PSI (Person Separation Index) ≥ 0.80 for reliability assessment by measurement scale, χ^2^ *P*-value > 0.05 [Bonferroni-adjusted], Items- and Persons- Fit Residual Mean ~ 0 and SD (Standard Deviation) ~ 1, less than 5 % significant *t*-test

*Significant after *P* < 0.05 with Bonferroni correction for the number of items in the analysis

^a^Had local item dependency

**Fig. 1:**
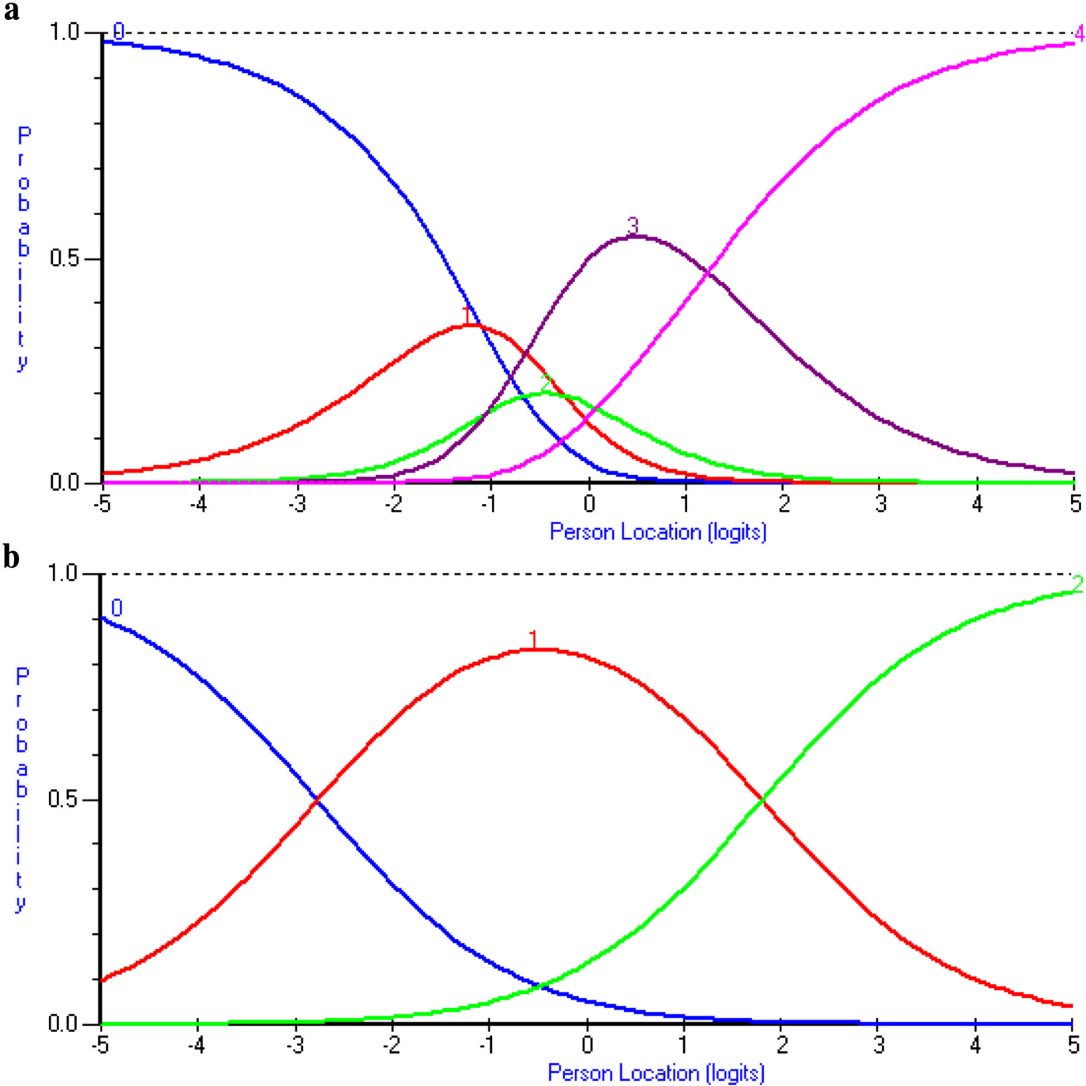
Category probability curves of one item from the modifying subscale - ‘I participate in certain activities less often to avoid aggravating my knees’ before formation of the testlets. Panel **a**: (Before Rescored) depicts disordered response category thresholds. Panel **b**: (After Rescored) depicts the item’s response scale after the three inner response categories were rescored to have an equal value of one, thus creating a logical and sequential ordering of its thresholds

Data of eight misfit persons were deleted. Eight items had disordered thresholds. There was equitable utilization of response categories across most items. The exceptions were items of the medications subscale, for which the subjects predominantly endorsed the ‘None – 0’ category. Rescoring the category response structure of all 13 items from five-level to three-level numeric response categories resolved all threshold disorder. In this new category response structure, the middle three response options have the same value (0-1-1-1-2), thus assigning an equal score for the three inner response categories. As an example, Fig. 1 depicts the category probability curves of one item of the modifying subscale before and after being rescored.

At this point, no individual item was a misfit. The data met summary fit criteria to the Rasch model and there was no DIF. However, the residual correlation matrix of the items indicated that the four subscales had local item dependency which grouped the items into their respective subscales. Only one pair of items of the interpreting subscale had residual correlations >0.2, but its items were still considered a testlet because their residuals were most correlated with each other. The results from Horn’s parallel analysis coupled with these results, suggested that the dominant construct in the QuIKS is a higher-order factor, while its subscales are lower-order factors. Existing theory, prior research and the preceding results in this study guided our decision to form four testlets corresponding to the original four subscales. There was a large proportion of common variance (A = 0.93) among the testlets, which indicated that a single dominant construct was captured by the QuIKS. After re-entering the data previously deleted for misfitted persons and making the preceding modifications to the QuIKS’s data, only data of four subjects were deleted; one with an individual data pattern that misfitted the Rasch model and three subjects with data missing for one item.

**Fig. 2:**
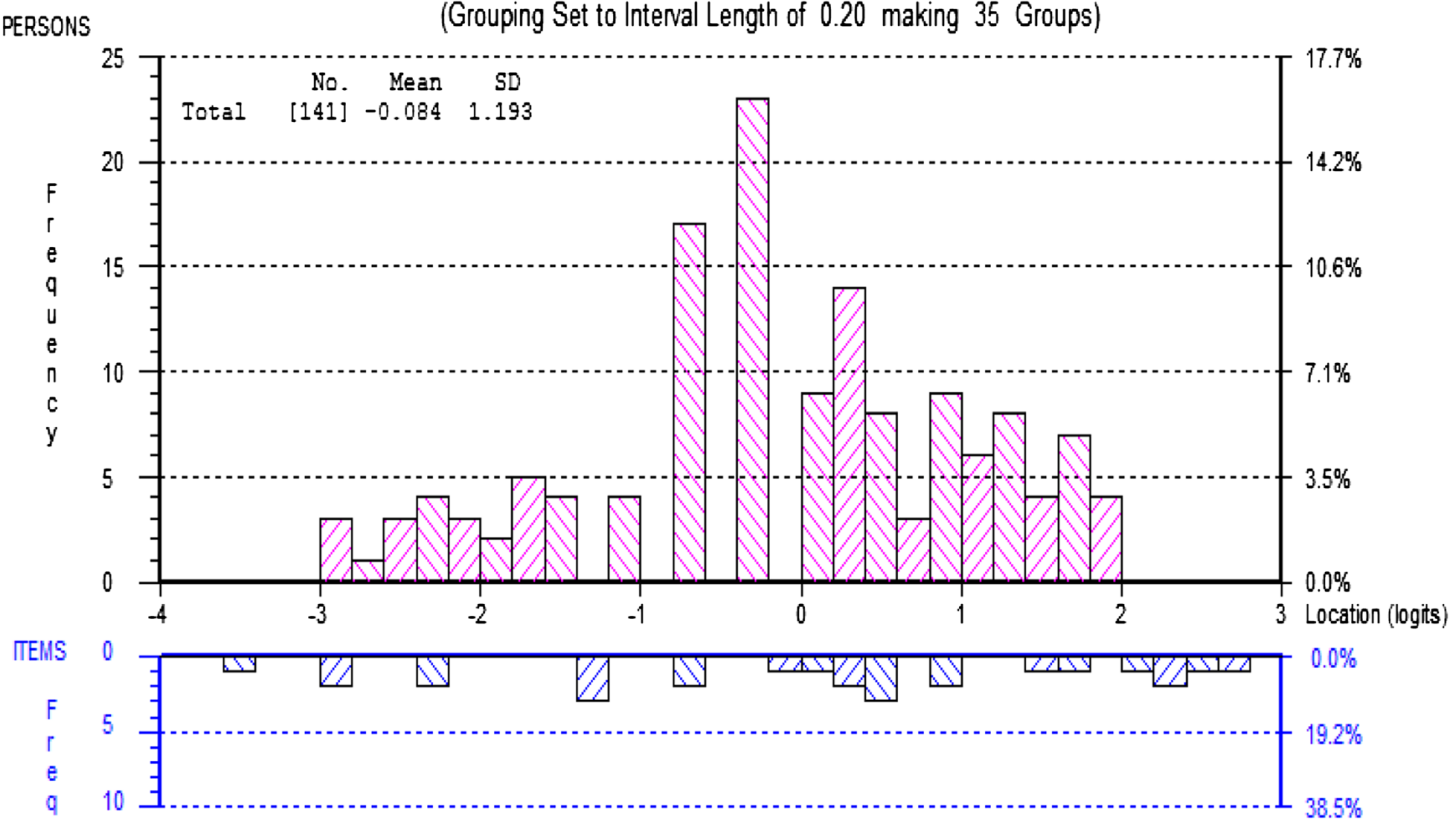
Fitting persons and items threshold distribution on the same logit scale. The distribution of the subjects’ estimate of knee symptoms-related experiences is in the upper histogram, with increasing levels of knee symptoms-related experiences from left to right on the x-axis. The lower histogram shows the distribution of the 13 items’ response categories threshold estimates, with higher levels of knee symptoms-related experiences from left to right on the x-axis

This refined QuIKS conformed to the expectations of summary fit to the Rasch model, as revealed by a non-significant item-trait interaction *χ*^2^, *see* Table 3. Only 3.0 % of subjects had significant independent *t*-tests, confirming the unidimensionality of the underlying construct in the refined QuIKS. This Rasch-validated QuIKS had a PSI of 0.82, which is adequate to distinguish up to three distinct levels of its underlying construct. Figure 2 depicts findings that suggested the Rasch-validated QuIKS was suitable for assessing the subjects, because the mean (SD) person estimate was 0.08 (1.19) with an item estimate mean of 0.00. The subscales of the Rasch-validated QuIKS had a hierarchical order from less to more knee symptoms-related experiences in logit scores as follows: monitoring (−0.886), modifying (−0.192), interpreting (−0.112) and medication (1.19). There were no floor or ceiling effects. The Additional file 1 provides the Rasch-validated QuIKS. A table at the bottom of the Rasch-validated QuIKS form provides the interval-level scores (vary 0 to 100) that correspond to the total raw scores (vary 0 to 26).

### Factor structure of QuIKS

Results from confirmatory factor analysis substantiated the results from the Horn’s parallel analysis and Rasch analysis. We tested the one-dominant construct (second order factor) and four-testlet (first order factors) structure of the 13-item Rasch-validated QuIKS, and the data showed adequate fit to the model [CFI = 0.94, TLI = 0.92, and RMSEA = 0.08 (95 % CI = 0.06–0.10]. Thus, the Rasch-validated QuIKS conformed to a unidimensional model.

### Known-groups validity

The Kruskal-Wallis H test, where H is the test statistic, revealed that the total scores on the Rasch-validated QuIKS were significantly different among the three knee health groups (H = 123.01, *df* = 2, and *P* < 0.001), with a median (inter-quartile range) of 100.0 (12.7) for HK, 52.9 (21.4) for KP, and 29.7 (13.8) for pre-HTO. There was a statistically significant moderate effect size between the HK and KP groups (*n* = 166) with Cohen’s *d* = 2.20 (95 % CI = 1.81, 2.60), *z* = −9.615, and *P* < 0.001, which indicated less knee symptoms-related experiences in the HK group compared to the KP group. There was a significant moderate effect size between the KP and pre-HTO groups (*n* = 145) with Cohen’s *d* = 1.32 (95 % CI = 0.99, 1.66), *z* = −6.641, and *P* < 0.001.

### Convergent validity

The QuIKS had statistically significant moderate correlation point estimates of *r*_*s*_ between 0.45 and 0.77 with each KOOS subscale. Its lowest correlation was with the KOOS-other symptoms (*r*_*s*_ = 0.45 [95 % CI = 0.31, 0.57]), followed by KOOS-sports and recreation function (*r*_*s*_ = 0.65 [95 % CI = 0.54, 0.74]), KOOS-activities of daily living (*r*_*s*_ = 0.70 [95 % CI = 0.60, 0.78]), KOOS-Pain (r_s_ = 0.72 [95 % CI = 0.63, 0.79]), and its highest correlation was with KOOS-quality of life (*r*_*s*_ = 0.77 [95 % CI = 0.69, 0.84]).

## Discussion

Our findings affirmed the hypotheses in this study. An updated version of the QuIKS, called the QuIKS-R, was adequately validated using information from Rasch analysis. The results suggest that the QuIKS-R encapsulates all four of its subscales into a unidimensional measure of experiences associated with early symptoms that are consistent with symptomatic knee OA. For clinicians and researchers, these findings mean that ratings on the QuIKS-R can be validly summed, much like marks on a ruler. First, calculate the total raw score, then use the conversion table at the bottom of the QuIKS-R (see Additional file 1) to obtain the corresponding interval-level (final) total score. These interval-level scores are an individual’s level of knee symptoms-related experiences. To the best of our knowledge, the QuIKS-R would be the first unidimensional measure designed to quantify experiences specifically associated with early symptoms of symptomatic knee OA [41, 42].

It made conceptual sense to condense the three middle response categories of each item, given the descriptors used for these categories. In the medication subscale we combined ‘Rarely’, ‘Sometimes’, and ‘Often’. We did this because it might have been difficult for subjects to recall their illness response and then choose a response category that best classified their experience. It is possible that subjects did not have a consistent pattern of selecting between ‘Rarely’ and ‘Sometimes’ and between ‘Sometimes’ and ‘Often’. Perhaps more clearly defined descriptors, for example, ‘Rarely = 1 to 3 times per week’, ‘Sometimes = 4 to 6 times per week’, and ‘Often = 7 to 9 times per week’ would remove ambiguity from among these categories [43]. Furthermore, the other 10 items used five-point Likert scales with ‘Neutral’as their midpoint. The rescoring of these items could be explained in the context of the long history of debate on the implication of midpoints in rating scales [44]. A midpoint, such as ‘Neutral’, is sometimes misinterpreted or selected in a biased way [44]. However, its removal might push some respondents to choose adjacent categories and reduce the reliability and validity of the measure [44]. Therefore, scoring the midpoint in the same manner as its adjacent categories was deemed a good solution for these two issues.

Based on the hierarchical order of the logit scores of the subscales (testlets), this study suggests that the level of knee symptoms-related experiences increased as individuals moved from the monitoring, to the modifying, then interpreting, and finally to the medication subscale. This pattern means that subjects tended to indicate higher ratings on the monitoring subscale compared to the medication subscale. This pattern fits with a grounded theory of experiences and behaviour that people with emergent chronic knee problems engage in to prevent damaging their knee, a theory called ‘Being Careful’ that describes “the process of recognising the onset of chronic knee problems” (p. 939) [5]. This pattern also fits with the model of illness behaviour which is a representation of the decision-making process during an illness [45]. This model employs nine stages, starting from illness recognition and labeling to the application of treatment with consequential re-evaluation of the illness state by the individual, in an iterative process [45]. Furthermore, the model of selective optimization with compensation [46] also offers a theoretical basis for why items from different subscales form a unidimensional construct in the QuIKS-R, as it provides an explanation of the process of adaptation in people with knee pain problems. For example, in the early stages of symptomatic knee OA, one would expect that a person might make the decision to stop engaging in a favorite activity because of their knee pain (selection), change their exercise routine because of the knee problem (compensation), and take medication before activity to prevent pain (optimization) [46, 47]. For clinicians these findings mean that scores on the QuIKS-R covers a continuum of knee symptoms-related experiences in people with knee symptoms that are consistent with symptomatic knee OA.

Forming testlets to obtain unidimensionality demonstrated that the subscales of the QuIKS are sub-constructs of a unified set of complex experiences in people with knee symptoms. When measuring a construct, measures with fewer items tend to have higher accuracy but lower precision [13, 14]. By forming the testlets, we were able to capitalize on the accuracy of the subscales, while capitalizing on the precision of the full questionnaire to provide more information about an individual’s level of knee symptoms-related experiences. It is worth noting that the individual testlets should not be used for score interpretation. Only total scores from all 13 items of the QuIKS-R should be interpreted, and this interpretation should be in the context of the higher-order construct of knee symptoms-related experiences.

The QuIKS-R discriminated between the study groups. The pre-HTO group had the highest level of knee symptoms-related experiences, followed by the KP, then the HK group, with a significant between-group difference of at least a moderate effect size. There are no previous studies of the QuIKS with which to compare these findings. However, population-based reference data of each subscale of the KOOS supports the values obtained in the present study [48, 49]. For example, the KOOS-pain median score for the KP and pre-HTO groups were 80.6 and 48.6 respectively, and 97.2 for people aged 35 to 54 years in a population-based group [48]. This is logical given that the prevalence of symptomatic knee OA increases with age and OA-related knee pain usually becomes more severe over time [3, 50]. A lower correlation between the QuIKS-R and the KOOS-other symptom subscale compared to the QuIKS-R correlation with the other KOOS subscales, could mean that the level of knee symptoms-related experiences in the study population was less related to other joint impairments but highly related to pain severity, activity limitations and knee-related quality of life. Nevertheless, the significant moderate correlations between the scores on the QuIKS-R and each subscale of the KOOS, suggest that there are important relationships between the constructs in the two measures. For clinicians, these findings could mean that the QuIKS-R may be useful in discouraging physical activity limitations while helpful in promoting or maintaining physical activity and quality of life in patients with knee symptoms consistent with symptomatic knee OA.

A major implication of the current study is that the QuIKS has now adequately achieved construct validation through creation of the QuIKS-R. Whereas the original QuIKS had ambiguity across the categories of each item’s response scale and was not unidimensional, the QuIKS-R is unidimensional and provides interval-level scores. These interval-level scores mean that equal unit differences along the QuIKS-R scale represent equal amounts of its underlying construct, regardless of where on the scale these differences occur. Overall the findings in the current study imply that the QuIKS-R has adequate discriminative ability. The QuIKS-R may be used as a discriminative tool but has not been validated as an evaluative measure. Also, whereas the original QuIKS is a “self-administered questionnaire used to promote activity by identifying the experiences associated with early symptoms consistent with knee OA” (p. 1) [9], the QuIKS-R shares this purpose by using a more refined scale.

## Limitations and future research

A limitation of this study was that the subjects in the KP group did not receive a medical diagnosis, so their knee pathology could be unrelated to knee OA. Also, while known-group (discriminative) validation supported the QuIKS-R ability to discriminate the level of knee symptoms-related experiences between healthy and two severely involved groups, this information might not be useful for a clinician’s assessment of individual patients. Future studies should use a larger sample and evaluate the predictive validity of the QuIKS-R in identifying subjects with OA-like knee pain who are at greatest risk for physical activity limitations.

## Conclusions

The QuIKS-R is a unidimensional measurement scale that provides interval-level scores of knee symptoms-related experiences in persons with knee symptoms consistent with symptomatic knee OA. Scores on the QuIKS-R that represent more knee symptoms-related experiences, also mean that a patient is more aware of and affected by their knee symptoms, and has tried more to remedy their condition. This information might be useful for clinicians when providing pain management interventions and for promoting activity in individuals with early symptoms consistent with symptomatic knee OA.

## Additional file

Additional file 1:
**Questionnaire to Identify Knee Symptoms-R (QuIKS-R).** (DOCX 17 kb)

## Abbreviations

A: Common variance among testlets
BMI: Body mass index
CFI: Comparative fit index
CI: Confidence interval
DIF: Differential item functioning
H: Test statistic for Kruskal-Wallis H test
HK: Pain-free healthy knees
KP: Knee pain with no knee osteoarthritis diagnosis
OA: Osteoarthritis
pre-HTO: Scheduled for high tibial osteotomy
PSI: Person separation index
QuIKS: Questionnaire to Identify Knee Symptoms
QuIKS-R: Rasch-refined questionnaire to identify knee symptoms
*r*: Effect size – Mann Whitney *U* test
RMSEA: Root-mean-square error of approximation
*r*_*s*_: Spearman’s rank correlation coefficient
TLI: Tucker-Lewis index
