# Supplementary material for: Validation of the Questionnaire to Identify Knee Symptoms (QuIKS) using Rasch analysis
Source: Health Qual Life Outcomes. 2015 Sep 29;13:157. doi: 10.1186/s12955-015-0358-6 (PMC4587900; doi:10.1186/s12955-015-0358-6)
Supplement: Additional file 1: — Questionnaire to Identify Knee Symptoms-R (QuIKS-R). (DOCX 17 kb) [file 12955_2015_358_MOESM1_ESM.docx]

**Additional File 1**

**Questionnaire to Identify Knee Symptoms-R (QuIKS-R)**

**Instructions**

Tick one box to answer each question. If you are unclear about how to answer a question, please give your best answer.

**Medications**

The following statements describe things you might do to manage your knee pain with medications.

Tick the box that best describes how often each statement applies to you in the last 2 weeks.

|  | **Never** | **Rarely** | **Sometimes** | **Often** | **Always** |
| --- | --- | --- | --- | --- | --- |
| 1. I take pills before I do some activities to prevent knee pain. | □_0_ | □_1_ | □_1_ | □_1_ | □_2_ |
| 2. I take pills after I do some activities to reduce knee pain. | □_0_ | □_1_ | □_1_ | □_1_ | □_2_ |
| 3. I carry pills with me just in case my knees start to hurt. | □_0_ | □_1_ | □_1_ | □_1_ | □_2_ |

**Monitoring**

The following statements describe how you may monitor your knee symptoms.

Tick the box that best describes your agreement with each of the following statements in the last 2 weeks.

|  | **Strongly**  **Disagree** | **Disagree** | **Neutral** | **Agree** | **Strongly**  **Agree** |
| --- | --- | --- | --- | --- | --- |
| 4. I notice knee pain when kneeling. | □_0_ | □_1_ | □_1_ | □_1_ | □_2_ |
| 5. My knees feel stiff after sitting or standing for long periods of time. | □_0_ | □_1_ | □_1_ | □_1_ | □_2_ |
| 6. My knees hurt after sitting or standing for long periods of time. | □_0_ | □_1_ | □_1_ | □_1_ | □_2_ |

(Continues)

**Interpreting**

The following statements describe how you may interpret your ongoing knee symptoms.

Tick the box that best describes your agreement with each of the following statements in the last 2 weeks.

|  | **Strongly**  **Disagree** | **Disagree** | **Neutral** | **Agree** | **Strongly**  **Agree** |
| --- | --- | --- | --- | --- | --- |
| 7. I talk to family and friends about things I can do about my knee problems. | □_0_ | □_1_ | □_1_ | □_1_ | □_2_ |
| 8. I consult my doctor about my knee problems. | □_0_ | □_1_ | □_1_ | □_1_ | □_2_ |
| 9. I suspect my knee problems are the result of getting older. | □_0_ | □_1_ | □_1_ | □_1_ | □_2_ |
| 10. I suspect my knee problems are arthritis. | □_0_ | □_1_ | □_1_ | □_1_ | □_2_ |

**Modifying**

The following statements describe how you may modify activities in response to knee pain.

Tick the box that best describes your agreement with each of the following statements in the last 2 weeks.

|  | **Strongly**  **Disagree** | **Disagree** | **Neutral** | **Agree** | **Strongly**  **Agree** |
| --- | --- | --- | --- | --- | --- |
| 11. I participate in certain activities less often to avoid aggravating my knees. | □_0_ | □_1_ | □_1_ | □_1_ | □_2_ |
| 12. I am considering stopping a favorite activity due to my knees. | □_0_ | □_1_ | □_1_ | □_1_ | □_2_ |
| 13. I am considering changing my exercise routine due to my knee problems. | □^0^ | □_1_ | □_1_ | □_1_ | □_2_ |

(Continues)

| **The conversion table below is for Clinicians Use Only**  **QuIKS-R** raw total score = ____________ **QuIKS-R** final score = ______________  **Conversion Table** | | | | | |
| --- | --- | --- | --- | --- | --- |
| Total Raw Score | Final Score | Total Raw Score | Final Score | Total Raw Score | Final Score |
| 0 | 100 | 9 | 66.2 | 18 | 29.7 |
| 1 | 94.8 | 10 | 59.2 | 19 | 26.7 |
| 2 | 90.6 | 11 | 52.9 | 20 | 23.8 |
| 3 | 87.3 | 12 | 48.2 | 21 | 20.9 |
| 4 | 84.4 | 13 | 44.5 | 22 | 17.9 |
| 5 | 81.6 | 14 | 41.2 | 23 | 14.9 |
| 6 | 78.6 | 15 | 38.3 | 24 | 11.4 |
| 7 | 75.3 | 16 | 35.4 | 25 | 6.6 |
| 8 | 71.2 | 17 | 32.6 | 26 | 0 |
